# Effects of a 12-week high-intensity interval training intervention on serum neurotransmitters and autonomic function in mild-to-moderate depression

**DOI:** 10.3389/fpsyg.2026.1861321

**Published:** 2026-08-31

**Authors:** Zange Lin, Fengxun Lin, Shihui Yang, YingYing Diao, Jiahui Jin, Meihua Su

**Affiliations:** 1 Psychological Counseling Center, Jimei University, Xiamen, Fujian, China; 2 Physical Education Institute, Jimei University, Xiamen, Fujian, China

**Keywords:** autonomic nervous system function, depression, exercise, heart rate variability, HIIT, neurotransmitters

## Abstract

**Background:**

Exercise is increasingly recognized as an effective adjunctive treatment for depression; however, the neurobiological mechanisms underlying its antidepressant effects remain incompletely understood. This study investigated whether a 12-week high-intensity interval training (HIIT) program could alleviate depressive symptoms by modulating peripheral neurotransmitter profiles and autonomic nervous system function in university students with mild-to-moderate depression.

**Methods:**

A total of 34 university students (18–25 years) with mild-to-moderate depression were enrolled and allocated to either an exercise group (EXD, *n* = 17) or a control group (CON, *n* = 17). Participants in the EXD group underwent a supervised 12-week HIIT intervention consisting of three 60-min sessions per week, while participants in the CON group continued to receive their usual clinical care without additional structured exercise training. Depressive symptoms were evaluated using the Beck Depression Inventory (BDI) and the Hamilton Depression Rating Scale (HAMD-17). Autonomic nervous system function was assessed by heart rate variability (HRV), and peripheral serum concentrations of serotonin (5-HT), brain-derived neurotrophic factor (BDNF), cortisol, and glutamate were measured before and after the intervention.

**Results:**

Compared with the CON group, participants in the EXD group exhibited significantly greater reductions in BDI and HAMD-17 scores, accompanied by marked increases in serum 5-HT and BDNF levels and significant decreases in cortisol and glutamate concentrations (*p* < 0.05). Both groups exhibited significant within-group improvements in time-domain HRV parameters, including SDNN, RMSSD and HF power. Low-frequency (LF) power increased significantly only within the EXD group; however, no significant between-group differences were observed for any HRV parameters, including LF power and the LF/HF ratio.

**Conclusion:**

A 12-week HIIT program effectively alleviated depressive symptoms and improved autonomic nervous system function in young adults with mild-to-moderate depression.

## 1 Introduction

Depression is one of the leading causes of disability worldwide and represents a major public health challenge (Malhi and Mann, 2018). According to statistics from the World Health Organization (WHO), approximately 322 million people worldwide suffer from depression, with a lifetime prevalence of 15–18%, making it one of the major public health issues today (Banyard et al., 2025). It is characterized by persistent low mood, anhedonia, cognitive dysfunction, and autonomic dysregulation, substantially impairing quality of life and increasing the risk of suicide (Bellón, 2024). Although pharmacotherapy and psychotherapy remain the mainstay treatments, their effectiveness is often limited in individuals with mild-to-moderate depression because of suboptimal efficacy, poor adherence, adverse effects, and limited accessibility (Brown et al., 2022).

Accumulating evidence indicates that exercise is an effective adjunctive intervention for alleviating depressive symptoms and improving psychological well-being (Catena-Dell’Osso et al., 2011). Experimental studies suggest that these benefits may involve coordinated regulation of monoaminergic neurotransmission, brain-derived neurotrophic factor (BDNF), hypothalamic–pituitary–adrenal (HPA) axis activity, glutamatergic signaling, and autonomic nervous system function (Garber et al., 2011). However, most previous studies have focused primarily on clinical symptom improvement, whereas the neurobiological mechanisms underlying the antidepressant effects of exercise remain incompletely understood. In particular, few human intervention studies have simultaneously evaluated changes in peripheral neurotransmitters and autonomic function following structured exercise training.

Therefore, the present study investigated the effects of a 12-week high-intensity interval training (HIIT) program on depressive symptoms, heart rate variability (HRV), and peripheral serum biomarkers, including serotonin (5-HT), brain-derived neurotrophic factor (BDNF), cortisol, and glutamate, in university students with mild-to-moderate depression. We hypothesized that HIIT would alleviate depressive symptoms by improving autonomic regulation and modulating neurotransmitter-related pathways, thereby providing mechanistic evidence supporting exercise as an adjunctive treatment for depression.

## 2 Methods

### 2.1 Study design

Participants were recruited through the Psychological Center of Jimei University. Eligible participants were university students aged 18–25 years who met the DSM-5 diagnostic criteria for a major depressive episode. Both sexes were eligible. The protocol was approved by the Academic Ethics Committee of Jimei University (No. JMU202501001).

Inclusion criteria were: (1) DSM-5 diagnosis of a major depressive episode (unipolar); (2) Beck Depression Inventory-II (BDI-II) score > = 15; (3) resting heart rate > 50 bpm; (4) age 18–25 years.

Exclusion criteria: Exclude individuals with organic mental disorders, psychoactive substance dependence, severe somatic diseases, other physical or mental conditions that are not conducive to exercise, or those who already have a regular and intense exercise habit.

Participants’ habitual physical activity was assessed using the International Physical Activity Questionnaire–Short Form (IPAQ-SF). Individuals who had engaged in structured exercise training during the previous 3 months were excluded to minimize the influence of prior exercise adaptation. Baseline physical activity levels were recorded for descriptive purposes, and no significant differences were observed between the two groups at baseline.

### 2.2 Sample size

This study was based on the assumption of comparing the means of two independent samples. *A priori* sample size estimation was performed using PASS 15.0 software. Based on the results of a preliminary experiment, the calculation was performed using the change in BDI-II score as the primary outcome measure (Ge et al., 2025), with a significance level *α* = 0.05 and a power (1−*β*) = 0.80. The calculated minimum sample size was 14 participants per group. Accounting for a 20% dropout rate, the final sample size was set at 17 participants per group, yielding a total of 34 participants.

### 2.3 Group assignment

Participants were randomly assigned (1:1) using a computer-generated random sequence created by an investigator not involved in recruitment. Allocation assignments were placed in sequentially numbered opaque sealed envelopes.

Control group (CON group): Participants continued their standardised treatment regimen received before enrolment without any additional structured exercise intervention.
Exercise group (EXD group): Participants underwent the exercise intervention while continuing their standardised treatment regimen received before enrolment.

All participants continued to receive their routine clinical care throughout the study. Pharmacological treatment and/or psychotherapy, when prescribed before enrollment, were maintained without modification during the 12-week intervention whenever possible. No new antidepressant medications or psychotherapy programs were initiated during the study period. Participants were instructed not to change their treatment regimen unless medically necessary, and any treatment changes were documented. Both the EXD and CON groups followed the same treatment principles, with the only difference being the additional HIIT intervention in the EXD group.

### 2.4 Exercise program

The exercise intervention was developed according to the FITT-VP principles recommended by the American College of Sports Medicine (ACSM) (Goodwin and Stein, 2021). Participants in the exercise group completed three supervised exercise sessions per week on non-consecutive days for 12 consecutive weeks, with each session lasting approximately 60 min (10-min warm-up, 40-min main exercise, and 10-min cool-down), resulting in a total exercise volume of approximately 180 min per week. The intervention consisted of three progressive phases.

#### 2.4.1 Adaptation phase (weeks 1–2)

Participants were familiarized with the exercise procedures and trained to safely perform the prescribed movements. The HIIT component consisted of 6 intervals of 30 s at 85–90% of HRmax interspersed with 30 s of active recovery at 60–70% HRmax. Resistance exercises included bodyweight squats, lunges, push-ups, and plank exercises (2 sets × 10 repetitions).

#### 2.4.2 Development phase (weeks 3–8)

Exercise intensity and volume were progressively increased. Participants completed 8–10 HIIT intervals performed at 90–95% HRmax with 30 s active recovery between intervals. Resistance training consisted of squats, lunges, push-ups, and core exercises performed for 3 sets of 8–10 repetitions at approximately 70–80% of one-repetition maximum (1RM), with 60–90 s rest between sets.

#### 2.4.3 Maintenance phase (weeks 9–12)

Participants maintained the prescribed training intensity and volume achieved during the development phase. HIIT consisted of 10–12 intervals at 90–95% HRmax separated by 30s active recovery. Resistance exercises remained unchanged to maintain neuromuscular adaptations.

Exercise intensity was continuously monitored using a Miopod heart-rate monitoring system (Suredoc Miopod Armband, Suredoc Health Technologies Co., Ltd., China) to ensure that participants maintained the prescribed heart-rate zones throughout each training session. All exercise sessions were supervised by certified exercise specialists, and attendance was recorded throughout the intervention. Participants were encouraged to attend all scheduled sessions, and attendance exceeding 85% was considered acceptable adherence.

Owing to the nature of the exercise intervention, participants and exercise instructors could not be blinded to group allocation. However, investigators responsible for outcome assessment, laboratory analyses, and statistical analyses remained blinded to group assignment throughout the study.

### 2.5 Outcomes

Surveys were conducted by uniformly trained and qualified investigators. The survey content mainly included: height, weight, BMI, smoking history, alcohol consumption history, sleep status, past medical history, basic family information, family history, medication history, etc. The short version of the International Physical Activity Questionnaire (reliability and validity Cronbach’s *α* = 0.81) was used to collect physical activity data from the past 7 days using a retrospective recall method (Okely et al., 2021).

#### 2.5.1 Anxiety and depression

Before and after the trial, the Beck Depression Inventory-II (BDI-II) and the Hamilton Depression Rating Scale (HAMD-17-17) were used to assess changes in mood status following the exercise intervention. Assessments were conducted by a professional registered psychological supervisor to ensure the accuracy of the mood status evaluation.

#### 2.5.2 Heart rate variability

Heart rate variability (HRV) is a non-invasive electrocardiophysiological indicator that quantifies the dynamic balance of the autonomic nervous system (ANS) by analysing fluctuations in consecutive heartbeat intervals, with its signal characteristics containing rich neuro-humoral-vascular interaction information (Junkes et al., 2025). Electrocardiographic signals were recorded for 5 min using a Finnish-made electrocardiograph (Mega Faros 180). Participants were instructed to avoid caffeine intake, vigorous exercise, and psychological stress before testing. After a 20-min resting period, participants were placed in a supine position and instructed to remain relaxed during recording. ECG signals were processed using the Pan–Tompkins algorithm for R-wave detection, followed by semi-automatic correction to remove artifacts and ensure accurate RR interval identification. HRV parameters, including the standard deviation of normal-to-normal intervals (SDNN), root mean square of successive differences (RMSSD), high-frequency power (HF), low-frequency power (LF), and LF/HF ratio, were calculated using Kubios HRV Standard software to evaluate autonomic nervous system function.

#### 2.5.3 Blood sampling and serum neurotransmitter detection

Before and after the intervention, fasting venous blood was collected from participants between 7:00 and 8:00 in the morning. After collecting 5 mL of blood using coagulation-promoting tubes, the samples were left to stand for 30 min, then centrifuged at 2000 rpm for 10 min to separate the serum. The separated serum was aliquoted into EP tubes, and enzyme-linked immunosorbent assay was used to measure serum levels of 5-hydroxytryptamine (5-HT), glutamate (GLU), cortisol, and brain-derived neurotrophic factor (BDNF).

### 2.6 Statistical analysis

Statistical analyses were performed using SPSS version 27.0 (IBM Corp., Armonk, NY, USA). Continuous variables are presented as mean ± standard deviation, whereas categorical variables are expressed as frequencies and percentages. Before conducting parametric analyses, the normality of continuous variables was assessed using the Shapiro–Wilk test, and homogeneity of variances was evaluated using Levene’s test. Baseline characteristics were compared between the EXD and CON groups using independent-samples *t*-tests for continuous variables and chi-square tests for categorical variables. Within-group changes from baseline to post-intervention were assessed using paired-samples *t*-tests, whereas between-group comparisons were performed using independent-samples *t*-tests. Effect sizes were calculated using Cohen’s *d* and Hedges’ *g*, and 95% confidence intervals were reported where appropriate to facilitate interpretation of the magnitude and precision of the intervention effects. All statistical tests were two-tailed, and exact *p* values are reported whenever possible. A *p* < 0.05 was considered statistically significant.

## 3 Results

### 3.1 Baseline characteristics of participants

**Figure 1:**
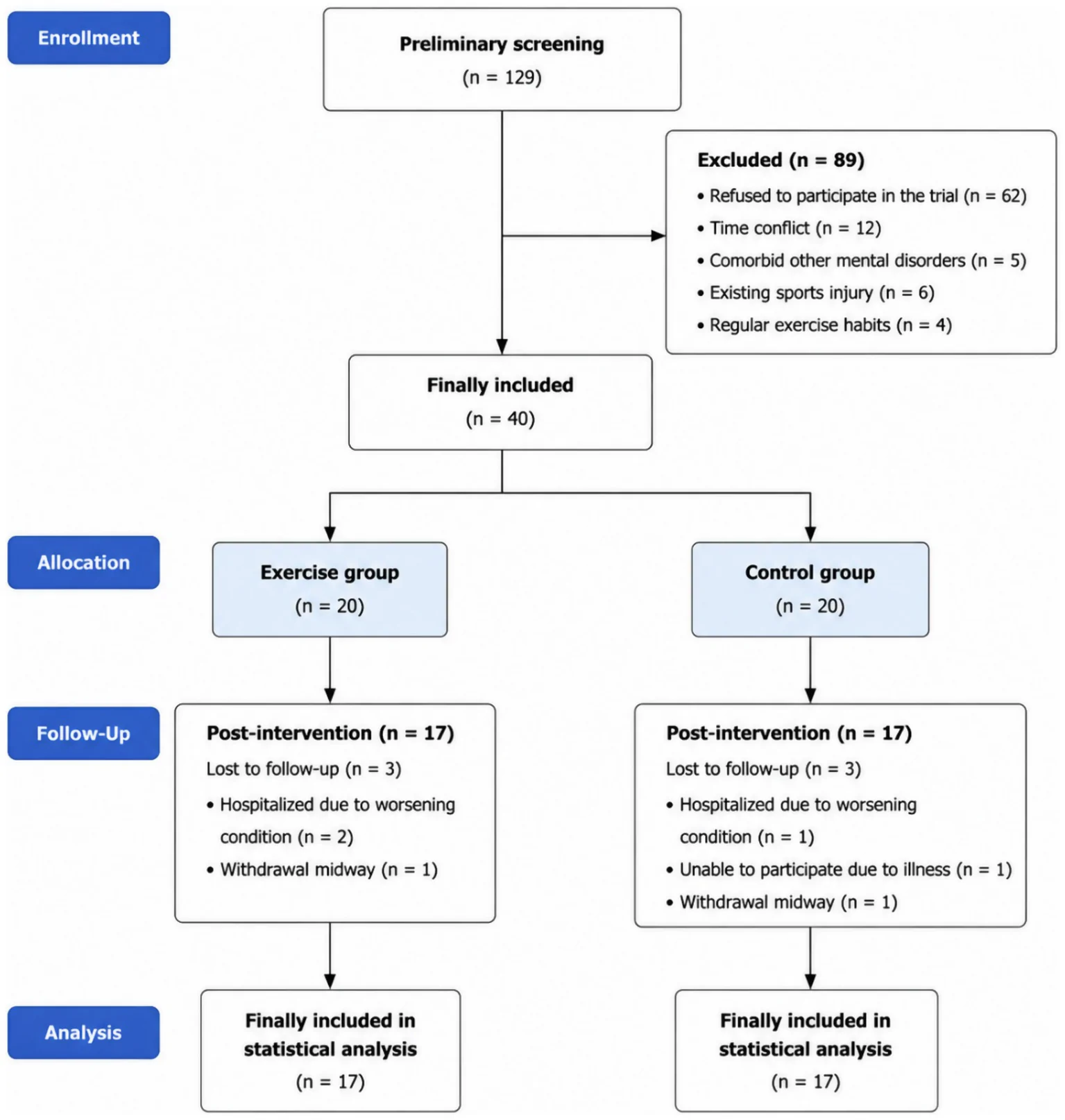
CONSORT flow diagram.

A total of 129 participants were screened, and 89 were excluded due to refusal to participate, time conflicts, comorbid psychiatric disorders, sports injuries, or regular exercise habits. Forty participants were finally enrolled and randomly assigned to the exercise group (*n* = 20) or control group (n = 20). After the intervention, three participants in each group withdrew, and 17 participants from each group completed the study and were included in the final statistical analysis (Figure 1); As shown in Table 1, there were no statistically significant differences between the two groups in terms of age, gender, height, weight, BMI, smoking, alcohol consumption (*p* > 0.05).

**Table 1:** Basic and demographic information of included participants.

Characteristics	Overall (*n* = 34)	CON (*n* = 17)	EXD (*n* = 17)	*t*-value	*p*-value
Age, year	18.94 ± 0.95	18.71 ± 0.77	19.18 ± 1.07	−1.47	0.152
Sex *N* (%)				–	0.296
Male	14 (41.18)	9 (52.94)	5 (29.41)		
Female	20 (58.82)	8 (47.06)	12 (70.59)		
Height, cm	168.21 ± 7.43	169.14 ± 8.07	167.28 ± 6.85	0.72	0.475
Weight, Kg	72.14 ± 10.31	72.51 ± 11.43	71.76 ± 9.40	0.21	0.836
Body mass index, kg/m^2^	25.40 ± 2.57	25.21 ± 2.62	25.59 ± 2.58	−0.43	0.671
Physical activity level (MET-min/week)	755.62 ± 546.99	817.47 ± 555.19	693.76 ± 548.39	0.65	0.518
Drinking, *n* (%)				–	1.000
Yes	4 (11.76)	2 (11.76)	2 (11.76)		
No	30 (88.24)	15 (88.24)	15 (88.24)		
Smoking, *n* (%)				–	1.000
Yes	8 (23.53)	4 (23.53)	4 (23.53)		
No	26 (76.47)	13 (76.47)	13 (76.47)		

### 3.2 The effect of exercise intervention on psychological and emotional changes in depressed participants

Shown in Table 2, prior to the exercise intervention, there was no significant difference in BDI and HAMD-17 scores between the EXD group and the CON group (*p* > 0.05), indicating that the baseline levels were consistent across both groups. Following the intervention, BDI and HAMD-17 scores in both groups decreased significantly compared to pre-intervention levels (*p* < 0.05). Further statistical analysis of the difference in scores between the two groups revealed that the EXD group performed significantly better than the CON group in terms of the difference between the two groups, suggesting that exercise intervention combined with conventional treatment is more effective than conventional treatment alone (Table 3).

**Table 2:** Changes in psychological and emotional states among participants with depression before and after the exercise intervention.

Indicators	Groups	Pre-	Post-	Difference (95% CI)	Cohen’s *d*	Hedges’ *g*	*t*-value	*p*-value
BDI	CON	20.47 ± 2.83	11.71 ± 3.51	8.765 (6.672, 10.857)	2.740	2.609	8.88	**<0.001**
	EXD	21.29 ± 3.33	8.82 ± 4.45	12.471 (10.625, 14.317)	3.079	2.933	14.32	**<0.001**
HAMD-17	CON	17.24 ± 2.02	10.00 ± 3.97	7.235 (5.395, 9.076)	2.144	2.042	8.33	**<0.001**
	EXD	18.06 ± 1.68	6.41 ± 3.36	11.647 (10.484, 12.810)	3.291	3.134	21.23	**<0.001**

Bold values indicate statistically significant differences (*p* < 0.05).

**Table 3:** Comparison of the differences in psychological and emotional states between the two groups of participants with depression before and after the exercise intervention.

Indicators	Groups	Difference	Difference (95% CI)	Cohen’s *d*	Hedges’ *g*	*t*-value	*p*-value
BDI	CON	−8.76 ± 4.07	3.706 (1.025, 6.387)	0.966	0.943	2.82	**0.008**
	EXD	−12.47 ± 3.59					
HAMD-17	CON	−7.24 ± 3.58	4.412 (2.320, 6.504)	1.473	1.438	4.30	**<0.001**
	EXD	−11.65 ± 2.26					

Bold values indicate statistically significant differences (*p* < 0.05).

### 3.3 Effects of exercise intervention on HRV parameters in depressed participants

As shown in Table 4, there were no significant differences in the time-domain (SDNN, RMSSD) or frequency-domain (LF, HF, LF/HF) indices between the two groups prior to the intervention, suggesting baseline comparability. Following the intervention, both SDNN and RMSSD increased significantly in both groups (*p* < 0.05). Regarding frequency-domain indices, LF increased significantly in the EXD group (*p* < 0.05), whereas no significant change was observed in the CON group (*p* > 0.05). HF increased significantly in both groups (*p* < 0.05), while the LF/HF ratio did not change significantly in either group (*p* > 0.05). Between-group differences in any HRV parameter (*p* > 0.05), indicating comparable overall improvements in heart rate variability between the two groups. Although the EXD group demonstrated a numerically greater increase in LF than the CON group, the between-group difference did not reach statistical significance, as shown in Table 5.

**Table 4:** Comparison of HRV parameters in participants with depression before and after the exercise intervention.

Indicators	Group	Pre-	Post-	Difference (95% CI)	Cohen’s *d*	Hedges’ *g*	*t*-value	*p*-value
SDNN (ms)	CON	16.72 ± 5.53	27.91 ± 13.05	−11.194(−19.060, −3.329)	−1.148	−1.093	−3.02	**0.008**
	EXD	24.25 ± 16.73	35.90 ± 21.62	−11.652 (−25.471, 2.167)	−1.602	−1.574	−1.79	**0.003**
RMSSD (ms)	CON	21.24 ± 12.32	58.88 ± 42.20	−37.647 (−60.152, −15.142)	−1.206	−1.149	−3.55	**0.003**
	EXD	35.71 ± 32.63	62.59 ± 47.11	−26.882 (−46.527, −7.238)	−0.635	−0.604	−2.90	**0.010**
LF (Hz)	CON	49.98 ± 21.20	61.73 ± 18.58	−11.749 (−25.602, 2.104)	−0.589	−0.561	−1.80	0.091
	EXD	45.31 ± 19.53	63.10 ± 17.63	−17.783 (−30.045, −5.521)	−0.955	−0.910	−3.07	**0.007**
HF (Hz)	CON	42.47 ± 17.04	57.83 ± 17.10	−15.355 (−24.421, −6.289)	−0.899	−0.857	−3.59	**0.002**
	EXD	40.37 ± 14.68	54.46 ± 19.50	−14.094 (−23.607, −4.581)	−0.804	−0.766	−3.14	**0.006**
LF/HF	CON	1.76 ± 1.21	1.25 ± 0.78	0.510 (−0.030, 1.050)	0.479	0.456	2.00	0.062
	EXD	1.89 ± 1.11	1.32 ± 0.61	0.564 (−0.128, 1.255)	0.633	0.603	1.73	0.103

Bold values indicate statistically significant differences (*p* < 0.05).

**Table 5:** Comparison of HRV differences between the two groups of participants with depression before and after the exercise intervention.

Indicators	Groups	Difference	Difference (95% CI)	Cohen’s *d*	Hedges’ *g*	*t*-value	*p*-value
SDNN (ms)	CON	11.19 ± 15.30	−0.458 (−15.737, 14.820)	−0.021	−0.020	−0.06	0.952
	EXD	11.65 ± 26.88					
RMSSD (ms)	CON	37.65 ± 43.77	10.765 (−17.939, 39.468)	0.262	0.256	0.76	0.451
	EXD	26.88 ± 38.21					
LF (Hz)	CON	11.75 ± 26.94	−6.034 (−23.810, 11.742)	−0.237	−0.232	−0.69	0.494
	EXD	17.78 ± 23.85					
HF (Hz)	CON	15.35 ± 17.63	1.261 (−11.366, 13.887)	0.070	0.068	0.20	0.840
	EXD	14.09 ± 18.50					
LF/HF	CON	−0.51 ± 1.05	0.054 (−0.789, 0.896)	0.044	0.043	0.13	0.898
	EXD	−0.56 ± 1.35					

### 3.4 Effects of exercise intervention on serum neurotransmitter levels in depressed participants

As shown in Table 6, there were no significant differences in the levels of 5-HT, GLU, cortisol and BDNF between the two groups prior to the intervention, indicating that the two groups were comparable at baseline. Following the intervention, levels of 5-HT and BDNF in peripheral serum were significantly higher in both groups compared to pre-intervention levels (*p* < 0.001), whilst GLU and cortisol levels were significantly lower (*p* < 0.001). Further analysis revealed that, for 5-HT and BDNF levels, the increase in the EXD group was significantly greater than that in the CON group (*p* < 0.001); for GLU and cortisol levels, the decrease in the EXD group was also significantly greater than that in the CON group (*p* < 0.001). Specific results are shown in Table 7.

**Table 6:** Comparison of serum neurotransmitter levels in depressed participants before and after the exercise intervention.

Indicators	Groups	Pre-	Post-	95% CI of difference	Cohen’s *d*	Hedges’ *g*	*t*-value	*p*-value
5-HT (ng/mL)	CON	153.56 ± 10.42	200.30 ± 15.41	−46.749 (−56.065, −37.433)	−3.548	−3.379	−10.64	**<0.001**
	EXD	145.75 ± 13.29	222.24 ± 18.61	−76.489 (−89.123, −63.856)	−4.748	−4.522	−12.83	**<0.001**
GLU (mg/L)	CON	10.93 ± 1.15	8.07 ± 0.67	2.854 (2.209, 3.499)	3.010	2.866	9.38	**<0.001**
	EXD	11.60 ± 1.23	7.52 ± 0.72	4.076 (3.356, 4.795)	4.032	3.840	12.01	**<0.001**
Cortisol (nmol/L)	CON	83.37 ± 7.52	66.49 ± 3.36	16.883 (13.396, 20.369)	2.653	2.527	10.26	**<0.001**
	EXD	87.10 ± 7.36	61.25 ± 3.57	25.849 (22.071, 29.628)	4.319	4.114	14.50	**<0.001**
BDNF (ng/mL)	CON	15.08 ± 1.72	19.38 ± 2.55	−4.305 (−5.460, −3.150)	−1.910	−1.819	−7.90	**<0.001**
	EXD	13.83 ± 2.22	22.44 ± 2.16	−8.610 (−10.309, −6.911)	−3.930	−3.742	−10.74	**<0.001**

Bold values indicate statistically significant differences (*p* < 0.05).

**Table 7:** Comparison of changes in serum neurotransmitter levels in depressed participants before and after the exercise intervention.

Indicators	Groups	Difference	95% CI of difference	Cohen’s *d*	Hedges’ *g*	*t*-value	*p*-value
5-HT (ng/mL)	CON	46.75 ± 18.12	−29.74 (−32.21, −27.27)	−1.378	−1.345	−4.02	**<0.001**
	EXD	76.49 ± 24.57					
GLU (mg/L)	CON	−2.85 ± 1.25	1.222 (0.293, 2.150)	0.919	0.898	2.68	**0.012**
	EXD	−4.08 ± 1.40					
Cortisol (nmol/L)	CON	−16.88 ± 6.78	8.966 (4.026, 13.907)	1.268	1.238	3.70	**<0.001**
	EXD	−25.85 ± 7.35					
BDNF (ng/mL)	CON	4.30 ± 2.25	−4.305 (−6.279, −2.331)	−1.524	−1.488	−4.44	**<0.001**
	EXD	8.61 ± 3.30					

Bold values indicate statistically significant differences (*p* < 0.05).

## 4 Discussion

The present study aimed to investigate the effects of a 12-week HIIT intervention on peripheral serum neurotransmitter levels and autonomic nervous system function in university students with mild-to-moderate depression. Throughout the intervention period, all participants continued their routine clinical management, including pharmacological treatment and/or psychotherapy when applicable, with treatment regimens maintained unchanged whenever possible. Therefore, the additional improvements observed in the HIIT group were more likely attributable to the exercise intervention rather than variations in concomitant treatments. Our findings demonstrated that HIIT not only significantly reduced depressive symptoms but also modulated the interplay among the monoaminergic system, HPA axis, and glutamatergic pathways, thereby improving autonomic nervous system regulation. These results provide further insights into the neurobiological mechanisms underlying the antidepressant effects of exercise.

Patients with depression have been reported to exhibit alterations in autonomic nervous system regulation, which may be associated with symptom severity and clinical outcomes (Carney et al., 2005). Heart rate variability (HRV) is a widely recognized, non-invasive indicator of autonomic nervous system function that reflects beat-to-beat fluctuations in the time intervals between consecutive heartbeats and provides valuable insights into autonomic regulation and cardiovascular function (Marasingha-Arachchige et al., 2022). HRV indices, including time-domain and frequency-domain parameters, have been commonly applied to evaluate changes in autonomic function in individuals with depression and following interventions (Gullett et al., 2023). Therefore, HRV analysis may provide an objective approach for assessing exercise-related changes in autonomic regulation (Wang et al., 2025). In the present study, SDNN, RMSSD, and HF increased significantly within both groups after the intervention, suggesting improvements in HRV parameters over time. However, no significant between-group differences were observed for any HRV indices. Although the EXD group showed a greater numerical increase in LF compared with the CON group, this trend did not reach statistical significance. Therefore, the observed HRV changes should be interpreted cautiously, and further studies with larger sample sizes are needed to clarify whether HIIT induces specific adaptations in autonomic regulation (Monroe and Harkness, 2022).

Monoaminergic system dysfunction is one of the pathogenetic mechanisms of depression (Perez-Caballero et al., 2019). Clinical studies have found that the concentration of monoamine neurotransmitters in the synaptic cleft of brain regions such as the prefrontal cortex, hippocampus, and limbic system is significantly reduced in patients with depression (Fitzgerald, 2024). The underlying mechanisms include: decreased tryptophan hydroxylase (TPH) activity leading to reduced synthesis of 5-HT (Alizadeh Pahlavani, 2024); Increased monoamine oxidase (MAO) activity may accelerate neurotransmitter degradation, while dysregulated tryptophan metabolism may shift toward the kynurenine pathway, potentially contributing to neuroexcitotoxicity (Okely et al., 2021). Long-term regular physical exercise upregulates TPH activity, promoting the biosynthesis of 5-HT (Deng et al., 2025); it also upregulates tyrosine hydroxylase (TH) expression, thereby enhancing the synthesis capacity of dopamine (DA) and norepinephrine (Philippot, 2022). This study found that exercise intervention significantly increased peripheral serum levels of 5-HT and BDNF while reducing cortisol concentration in depressed participants. The increase in serum 5-HT levels may reflect alterations in serotonergic regulation induced by exercise. Previous studies have suggested that exercise may influence serotonin metabolism through pathways involving monoamine oxidase activity and tryptophan hydroxylase regulation, although these mechanisms were not directly assessed in the present study. Similarly, the observed increase in BDNF levels may indicate enhanced neurotrophic signaling, which has been proposed as a potential contributor to exercise-related improvements in mood regulation and neuroplasticity (Ross et al., 2023). Furthermore, dysfunction of the glutamatergic system has been implicated in the pathophysiology of depression (Colucci-D’Amato et al., 2020). In the present study, exercise intervention significantly reduced serum glutamate levels, with a greater reduction observed in the EXD group compared with the CON group. This finding is consistent with previous studies suggesting that exercise may contribute to the maintenance of glutamate homeostasis. Potential mechanisms proposed in previous research include the regulation of BDNF-related signaling pathways and modulation of glutamatergic transmission; however, these pathways require further experimental validation (Watanabe et al., 2024).

## 5 Conclusion

The present findings suggest that a 12-week HIIT program may contribute to improvements in depressive symptoms, autonomic function, and peripheral neurotransmitter profiles in young adults with mild-to-moderate depression.

## 6 Study limitations

Several limitations of this study should be acknowledged. First, the relatively small sample size and recruitment from a single university may limit the generalizability of the findings to broader populations. Second, peripheral serum biomarkers may not fully reflect neurotransmitter activity and neurobiological processes within the central nervous system. Third, although the 12-week intervention period was sufficient to observe short-term physiological and psychological changes, the lack of long-term follow-up precludes assessment of the durability of exercise-induced benefits. Finally, dietary intake and habitual physical activity outside the supervised intervention were not objectively monitored, which may have introduced residual confounding effects. Future multicenter randomized controlled trials with larger sample sizes, long-term follow-up, objective monitoring of lifestyle behaviors, and comprehensive mechanistic biomarkers are warranted to further elucidate the biological pathways underlying the antidepressant effects of exercise.

## Data Availability

Publicly available datasets were analyzed in this study. This data can be found at: the original contributions proposed in this study are included in the article. Further inquiries can be directed to the corresponding author.

## References

[ref1] Alizadeh Pahlavani H. (2024). Possible role of exercise therapy on depression: effector neurotransmitters as key players. Behav. Brain Res. 459:114791. doi: 10.1016/j.bbr.2023.114791, 38048912

[ref2] Banyard H. Edward K.-L. Garvey L. Stephenson J. Azevedo L. Benson A. C. (2025). The effects of aerobic and resistance exercise on depression and anxiety: systematic review with meta-analysis. Int. J. Ment. Health Nurs. 34:e70054. doi: 10.1111/inm.70054, 40432290 PMC12117297

[ref3] Bellón J. Á. (2024). Exercise for the treatment of depression. BMJ 384:q320. doi: 10.1136/bmj.q320, 38355168

[ref4] Brown R. L. Chen M. A. Paoletti J. Dicker E. E. Wu-Chung E. L. LeRoy A. S. . (2022). Emotion regulation, parasympathetic function, and psychological well-being. Front. Psychol. 13:879166. doi: 10.3389/fpsyg.2022.879166, 35992409 PMC9381823

[ref5] Carney R. M. Freedland K. E. Veith R. C. (2005). Depression, the autonomic nervous system, and coronary heart disease. Psychosom. Med. 67, S29–S33. doi: 10.1097/01.psy.0000162254.61556.d5, 15953797

[ref6] Catena-Dell’Osso M. Bellantuono C. Consoli G. Baroni S. Rotella F. Marazziti D. (2011). Inflammatory and neurodegenerative pathways in depression: a new avenue for antidepressant development? Curr. Med. Chem. 18, 245–255. doi: 10.2174/092986711794088353, 21110802

[ref7] Colucci-D’Amato L. Speranza L. Volpicelli F. (2020). Neurotrophic factor BDNF, physiological functions and therapeutic potential in depression, neurodegeneration and brain cancer. Int. J. Mol. Sci. 21:7777. doi: 10.3390/ijms21207777, 33096634 PMC7589016

[ref8] Deng B. Di W. Long H. He Q. Jiang Z. Nan T. . (2025). The involvement of 5-HT was necessary for EA-mediated improvement of post-stroke depression. Transl. Psychiatry 15:382. doi: 10.1038/s41398-025-03621-y, 41053079 PMC12501360

[ref9] Fitzgerald P. J. (2024). Frontal alpha asymmetry and its modulation by monoaminergic neurotransmitters in depression. Clin. Psychopharmacol. Neurosci. 22, 405–415. doi: 10.9758/cpn.23.1138, 39069680 PMC11289606

[ref10] Garber C. E. Blissmer B. Deschenes M. R. Franklin B. A. Lamonte M. J. Lee I.-M. . (2011). American college of sports medicine position stand. Quantity and quality of exercise for developing and maintaining cardiorespiratory, musculoskeletal, and neuromotor fitness in apparently healthy adults: guidance for prescribing exercise. Med. Sci. Sports Exerc. 43, 1334–1359. doi: 10.1249/MSS.0b013e318213fefb, 21694556

[ref11] Ge H. Si L. Li C. Huang J. Sun L. Wu L. . (2025). The antidepressant effect of resveratrol is related to neuroplasticity mediated by the ELAVL4-bdnf mRNA pathway. Int. J. Mol. Sci. 26:1113. doi: 10.3390/ijms26031113, 39940881 PMC11817429

[ref12] Goodwin G. M. Stein D. J. (2021). Generalised anxiety disorder and depression: contemporary treatment approaches. Adv. Ther. 38, 45–51. doi: 10.1007/s12325-021-01859-8, 34417991 PMC8437834

[ref13] Gullett N. Zajkowska Z. Walsh A. Harper R. Mondelli V. (2023). Heart rate variability (HRV) as a way to understand associations between the autonomic nervous system (ANS) and affective states: a critical review of the literature. Int. J. Psychophysiol. 192, 35–42. doi: 10.1016/j.ijpsycho.2023.08.001, 37543289

[ref14] Junkes L. Quagliato L. A. Appolinario J. C. Shader R. I. Nardi A. E. (2025). MAO inhibitors for treatment-resistant depression: bringing an updated perspective on pioneering drugs. Pharmacol. Res. 219:107876. doi: 10.1016/j.phrs.2025.107876, 40712763

[ref15] Malhi G. S. Mann J. J. (2018). Depression. Lancet (Lond. Engl.) 392, 2299–2312. doi: 10.1016/S0140-6736(18)31948-2, 30396512

[ref16] Marasingha-Arachchige S. U. Rubio-Arias J. Á. Alcaraz P. E. Chung L. H. (2022). Factors that affect heart rate variability following acute resistance exercise: a systematic review and meta-analysis. J. Sport Health Sci. 11, 376–392. doi: 10.1016/j.jshs.2020.11.008, 33246163 PMC9189698

[ref17] Monroe S. M. Harkness K. L. (2022). Major depression and its recurrences: life course matters. Annu. Rev. Clin. Psychol. 18, 329–357. doi: 10.1146/annurev-clinpsy-072220-021440, 35216520

[ref18] Okely A. D. Kontsevaya A. Ng J. Abdeta C. (2021). 2020 WHO guidelines on physical activity and sedentary behavior. Sports Med. Health Sci. 3, 115–118. doi: 10.1016/j.smhs.2021.05.001, 35782159 PMC9219310

[ref19] Perez-Caballero L. Torres-Sanchez S. Romero-López-Alberca C. González-Saiz F. Mico J. A. Berrocoso E. (2019). Monoaminergic system and depression. Cell Tissue Res. 377, 107–113. doi: 10.1007/s00441-018-2978-8, 30627806

[ref20] Philippot A. (2022). Impact of physical exercise on depression and anxiety in adolescent inpatients: a randomized controlled trial. J. Affect. Disord. 301, 145–153. doi: 10.1016/j.jad.2022.01.011, 35007642

[ref21] Ross R. E. VanDerwerker C. J. Saladin M. E. Gregory C. M. (2023). The role of exercise in the treatment of depression: biological underpinnings and clinical outcomes. Mol. Psychiatry 28, 298–328. doi: 10.1038/s41380-022-01819-w, 36253441 PMC9969795

[ref22] Wang Z. Zou Y. Liu J. Peng W. Li M. Zou Z. (2025). Heart rate variability in mental disorders: an umbrella review of meta-analyses. Transl. Psychiatry 15:104. doi: 10.1038/s41398-025-03339-x, 40155386 PMC11953273

[ref23] Watanabe D. K. Jarczok M. N. Williams D. P. Koenig J. Thayer J. F. (2024). Evaluation of low vagally-mediated heart rate variability as an early marker of depression risk. J. Affect. Disord. 365, 146–154. doi: 10.1016/j.jad.2024.08.051, 39154979

